# Examining Sleep Quality in Adult Foster Care Alumni: Implications for Later Life Health and Well-Being

**DOI:** 10.3390/healthcare13141694

**Published:** 2025-07-15

**Authors:** Amanda Keller, Varda Mann-Feder, Delphine Collin-Vézina, Michael J. MacKenzie

**Affiliations:** 1 School of Social Work, McGill University, Montreal, QC H3A 1B9, Canada; 2 Department of Applied Human Sciences, Concordia University, Montreal, QC H3G 1M8, Canada; varda.mann-feder@concordia.ca; 3 Department of Pediatrics, School of Social Work, McGill University, Montreal, QC H3A 1B9, Canada; delphine.collin-vezina@mcgill.ca

**Keywords:** sleep quality, foster care alumni, care-leavers, well-being

## Abstract

**Background:** Foster care alumni face increased health challenges across the domains of mental and physical health, yet there is a paucity of research examining the associations between care experiences, health, and sleep quality in alumni aged 30 and above. **Objectives**: Our exploratory mixed-method study examined the sleep quality of North American group care leavers aged 30+ to understand whether sleep quality in adulthood is associated with earlier child welfare system experiences during childhood and adolescence. Secondly, we examined the association between sleep quality and overall concurrent health. **Methods**: Using a convenience sample of 41 alumni of care aged 30–85 and 16 qualitative interviews, we explored the intricate connections between group care leavers’ developmental trauma, sleep quality, and health. Linear regression and qualitative content analysis were utilized to understand how sleep was related to well-being in aging care alumni. **Results**: Adult sleep was significantly associated with the perceived quality of their youth out-of-home placement experiences (β = 0.421, *p* < 0.01), controlling for friendship support networks and demographic variables. Adult sleep quality was a significant predictor of overall health (β = −0.328, *p* < 0.05). Qualitative interviews elucidated insights into the importance and linkages of child welfare system experiences, adult sleep, and well-being. **Conclusions**: Our research highlights the enduring association between child welfare placement experiences, and sleep functioning well into adulthood, even when accounting for contemporaneous social support and other demographic indicators. Practitioners should be inquiring directly about sleep, and future longitudinal research should delve deeper into the nature of sleep difficulties and their association with health and well-being.

## 1. Introduction

The association between unresolved trauma experiences and elevated rates of sleeping difficulties is well established for the general population [1,2,3]. The literature is increasingly making the case that sleep challenges play a critical role in shaping health trajectories, with links to obesity [4,5], diabetes [6,7,8], and cardiovascular disease [9,10,11], amongst other conditions. Given this cutting-edge work on the importance of sleep to developmental well-being, a surprising paucity of research exists examining the connections between lived experience in child welfare placements and sleep, particularly for those with group or congregate care experiences, and its potential implications for lifelong health and well-being in adult care leavers.

A limited amount of the literature documents that youth in family-based foster care may experience increased sleeping difficulties while in care and that these sleeping difficulties are associated with both trauma exposure and mental health [12,13]. A higher incidence of sleeping challenges appears to occur in former foster youth emerging into the adulthood period [14]. Sleep has the potential to impact multiple domains of well-being, including health [15], mental health [16], and social relationships [17], in an amplifying bidirectional or transactional manner [18]. Sleep challenges can, thus, serve as an upstream developmental predictor of later challenges, as well as a downstream developmental effect of earlier mental health difficulties [19], and greater attention to sleep functioning may help inform developmentally informed interventions.

### 1.1. Literature Review

Sleep has been established as a critical component of global health and well-being [20], with good sleep quality associated with better health, mental health, and social relationships [21,22]. It has also been established that people who have unresolved trauma or loss sleep less well than the general population [1,2,23]. A nascent body of the literature demonstrates that foster children currently in placement do not sleep as well as their same-aged peers [12,24,25]. Female foster children and those with more interpersonal trauma exposure appear to experience disproportionately worse sleep [13,26], and some evidence suggests that certain foster care placement conditions may be associated with better sleep [25].

### 1.2. Sleep and Foster Care Alumni

As foster children transition to adulthood, sleep challenges appear to continue. One Flemish study reported insomnia symptoms in 40% of foster care alumni, compared to 26.4% of the general population [27]. Sleep appears to be influenced by anxiety in this population, as those with higher post-care anxiety levels also report higher rates of sleep disturbances [28]. This anxiety-related finding is supported by another study of care leavers which found that those who reported more intrusive traumatic memories experienced greater difficulties sleeping [29]. These three studies are among the first that have studied care leaver sleep quality. They focused, however, exclusively on transition-aged participants aged up to 27. Understanding these trajectories better in older adult care leavers may help inform interventions and support for care leavers’ needs as these adults age.

The purpose of this exploratory mixed-method study involving North American group care leavers was to explore the connections between developmental trauma, sleep, and psychosocial function. Specifically, to address the gap in understanding around the associations between group care experiences and sleep, we explored the following: (1) how the sleep quality of this population is predicted by the perceived quality of youth placement experiences, and (2) the associations between sleep quality and health in middle and later adulthood.

## 2. Materials and Methods

### 2.1. Participants

Participants were recruited via convenience sampling seeking those with experience living in out-of-home child care placements in a group or congregate care setting. Flyers for the study were posted on online Facebook groups of foster care alumni networks, and they were sent out via newsletters to current and retired child welfare system staff. Between 1 June 2022 and 26 December 2023, 55 people clicked on the link for the survey. A handful did not complete any questions, and 9 completed only a few questions. In order to reduce nonresponse bias and improve data quality, we set exclusion criteria for individuals who did not complete at least 70% of the survey’s questions, leaving a sample of 41 survey participants, 16 of whom went on to participate in an hour-long semi-structured narrative interview. Participants were aged 30–85. The participants were 85% Canadian and 15% American.

The study underwent an ethics review and received approval from the Ethics Board at McGill University (approval number 22-02-002), including approval of the informed consent process for both the survey and the qualitative interview. All survey participants provided informed consent, and all who went on to complete the subsequent qualitative interview provided further informed consent for the interview. The consent forms are linked to the manuscript.

### 2.2. Assessments and Measures

Participants consented online using Qualtrics (Provo, UT, USA) to complete a demographics survey that included questions about their youth protection experiences. They were given the following normed survey measures: a sleep quality scale [30], the Lubben Social Network Scale Revised-6 [31], and six questions from the Short Form Health Survey SF-8 [32]. These measures were selected for both their brevity and normed reliability. The sleep quality scale is a well-established single-question 10-point item which is used to measure sleep quality [30]. The Lubben Social Network Scale Revised-6 [31] is a six-item self-report device to measure social engagement, including interactions with family and friends, which produces scores between 0 and 30 that are used to assess social isolation in adults. The measurement incorporates both quantity (size of network) and quality aspects (closeness and frequency of contact) of social support. The SF-8 [32] is a widely used instrument for measuring health-related quality of life that derives from the longer SF-36, taking one question from each of the SF-36’s eight domains of health functioning. The SF-8 provides a global indicator of overall health including components of physical and mental health.

### 2.3. Demographic Variables and Frequencies

Descriptive data were analyzed using IBM SPSS (version 29.0.1.1), and we report on the frequencies of age, gender, race, ethnicity, placement length, and placement location in Table 1.

### 2.4. Statistical Analysis

Linear regression was used to examine the association between youth care placement quality and current sleep quality. We then examined each participant’s sleep, placement quality, and health, and also entered covariates for their current friendship support network, age, gender, race, ethnicity, education, and income. To determine why sleep is important, linear regression was also used to predict care leavers’ health using sleep and social networks, as well as age, gender, race, ethnicity, education, and income.

### 2.5. Qualitative Interview

Of the 41 people who completed the screening survey, 25 consented to be contacted for part two of the study, a qualitative interview. A total of 4 were excluded before making contact, because they reported less than two years in group care settings. Additionally, 1 person’s email was not valid and 2 people declined to participate. In total, 16 people were interviewed, 3 in person and 13 over the online platform Zoom.

All interview participants provided informed consent prior to the interview. We asked participants to share their out-of-home care story, and to share their experiences from their transition to adulthood and parenthood. We designed these questions with a feminist intersectional [33] and ecological life course [34] framework. The life course narrative interview had no questions specific to sleep, yet sleep-related themes did emerge during the interview process as some participants reported, unprompted, that their sleep affected their lives.

Interviews took between 45 min and two hours. Most interviews were completed in one day. Two people had interviews spread over two days due to childcare obligations. No one withdrew before or during the interview. Data was anonymized and pseudonyms are utilized in the paper.

### 2.6. Analysis

Based on the statistically significant findings related to sleep quality, we employed content analysis and reviewed the transcripts for any mention of sleep in the qualitative interviews. We specifically searched all transcripts for any mention of ‘dream’, ‘nightmare’, ‘sleep’, ‘fatigue’, ‘rest’, and ‘tired’. There were eight interviews which mentioned one of these words and of those, four were selected to share here in this article. All names or locations mentioned in these quotes are pseudonyms, which were often selected by participants during member checking.

### 2.7. Ethical Considerations

This research protocol was ethically approved by the McGill Ethics Research Board, REB File number 22-02-002. The initial screening survey focused on documenting demographics, health, social relationships, and sleep. It did not ask any probing questions about trauma or loss. The qualitative interviews were open-ended. During the consent process we specifically told participants that we were not collecting trauma stories, that they were welcome to share any details of their stories including traumatic events, but that we were most interested in their lives now and insights for improving child welfare in the future. Participants were reassured that they could refrain from engaging in any uncomfortable conversations by stopping or pausing the interview or simply changing the topic. The interviewer and primary researcher used a peer interview approach [35], as she is a lived experience researcher. Most participants reported a positive experience with the interview process during member-checking.

## 3. Results

### 3.1. Demographics

The study involved a diverse group of participants. In terms of gender, 16 participants identified as men, 23 as women, and 2 as non-binary or third gender. Regarding racial identity, 30 identified as white, 10 identified as racialized or Indigenous. A total of 7 reported some high school education, 4 had received a high school diploma or GED, 9 had received a post-secondary certificate or diploma, and 21 had attained a bachelor’s degree or higher. Detailed participant demographics are available in Table 1.

### 3.2. Sleep Predictors

Table 2 presents a series of linear regression analyses, with Model 1 demonstrating a significant positive relationship between adult sleep and perceived quality of youth protection experiences (β = 0.505, *p* < 0.01). Model 2 demonstrated a significant positive relationship between the quality of sleep and the Lubben friendship support network subscale (β = 0.360, *p* < 0.05). In Model 3 of Table 2, we found that the quality of youth protection remained a significant (β = 0.421, *p* < 0.01) predictor of sleep, even when considering the contemporaneous friendship support network. In Model 4, after controlling for friendship support network, age, gender, race, ethnicity, education, and income, only the quality of youth protection experiences remained significant (β = 0.549, *p* = 0.004), with the full model accounting for 23% of the variance in sleep quality. This suggests that the perceived quality of a placement experience predicts sleep quality decades after the placement, with effects that cannot be buffered by more temporally proximal markers such as the size and quality of the current social relationship network.

### 3.3. Health Predictors

To assess health, we employed six questions from the short-form health survey covering dimensions of both physical and mental health. This abbreviated subscale shows strong internal reliability, with a Cronbach’s Alpha coefficient of 0.89. Table 3 presents the results of a series of regression analyses, with Model 1 demonstrating that sleep is a significant predictor of overall health (β = −0.451, *p* < 0.01), with better sleep being associated with fewer health problems. In Model 2, we examined the association between friendship support networks and health, finding that higher support is associated with better health (β = −0.579, *p* < 0.001). In Model 3, we found that sleep (β = −0.279, *p* = 0.06) significantly predicts health problems, even after considering friendship support (β = −0.477, *p* < 0.01). Finally, in Model 4, we found that sleep (β = −0.328, *p* < 0.05) remains a significant predictor of health, even after controlling for friendship network, age, gender, race, ethnicity, marital status, and income, with the full model accounting for 54% of the variance in reported health problems.

### 3.4. Qualitative Findings

The interview did not probe the participants specifically for information on sleep; however, the following unprompted testimonials elicited from questions about care stories, transitions from care and to parenthood, and their life now emphasize the intricate connection between sleep, health, and child welfare experiences.

*Stormy*, age 40, reflected on her persistent sleep struggles that haunted her for years after leaving group homes. She articulated a restless state marked by chronic sleep deprivation.

*After group homes, I barely slept. There were years of a few hours of sleep a night. And it wasn’t necessarily because I couldn’t sleep like I was having a bad dream. Well, yeah, it was because I couldn’t sleep, but it wasn’t like I was having bad dreams or anything like that. It was just more along the lines of I just couldn’t get my body to rest.*

*Brooke*, age 50, in contrast, reflected on the enduring impact of her time in group homes as she still struggles with group home nightmares.

*I would say that it’s so weird because I’m 50 now and I still have group home dreams. If I do something to make a co-worker mad, or if I have an argument with my husband, I’ll have a nightmare that night that I’m being sent back to a group home.*

*Leanne*, age 40, delving into her insomnia and its repercussions, narrated how her struggle with fatigue during her early twenties culminated in a life-altering event.

*I wasn’t on Concerta, and I really believe that my ADHD and being tired all the time, like, my whole BSW is falling asleep in my books. And I fell asleep driving [and was] in a car crash that year. So that was like 2014. So, I have some physical stuff from that.*

Conversely *Tony*, age 69, revealed a more unique intersection of grief, insomnia, and substance use or self-medication, highlighting how sleep disturbances can become intertwined with coping mechanisms.

*When Elanor died, my wife ended up getting a brain tumour, it was very sudden, and we thought she had an earache…. I started to have morphine in the house. I started to do more things, to try to sleep and didn’t realize that I was getting addicted to it. Because as a person, I never really had any experience with opiates.*

These narratives, shared by care leavers aged 40–69, underscore the intricate relationship between tumultuous experiences within the child welfare system and the enduring sleep challenges faced by some care leavers during adulthood. As we navigate through these personal accounts, with their unprompted reflections on the importance of sleep, it becomes apparent that the consequences of disrupted sleep extend beyond the immediate post-care period into later life.

## 4. Discussion

The results of this study are consistent with what is currently known about trauma history and its impact on sleep across the life course in the general population [2,36]. However, to our knowledge, this paper is the first that examines sleep quality in care leavers over age 30. Furthermore, this paper illuminates a crucial aspect of well-being that is under-examined in foster care alumni, insofar as negative perceptions of child welfare experiences are associated with lower sleep quality decades after placement. While it is not possible to report on causality or the direction of effects in what are likely complex bidirectional processes, this exploratory study provides valuable insights into the intersection of adverse childhood experiences and adult well-being in care leaver adults in middle age and later life. Children with acute or chronic adversity in out-of-home placement may experience long-term consequences during their lives. Thus, attunement to sleep [12,25], social support networks, and quality of placement matches [37] should be prioritized for children in care and tracked in aging care leavers.

In the qualitative component of this article, the last two participants mentioned critical health consequences related to their sleeping difficulties. We cannot be sure whether *Tony* or *Leanne’s* later life challenges were influenced by their childhood stresses, but both arrays of challenges appear to point to important potential points of entry for intervention that could have been missed earlier in life. Leanne reported falling asleep in class and driving often before her car accident in her twenties, indicating the potential importance of clinicians asking about sleep challenges and fatigue as part of earlier assessments for care leavers. Similarly, Tony’s addictions only began later in life when he was offering palliative care to his wife and found himself unable to sleep. This is suggestive of additional potential ports of entry, as physicians or nurses offering end-of-life care could inquire more fully about how family caregivers are doing, including asking about stress and sleep. When working with people undergoing pivotal transitions, clinicians need to ask about sleep, particularly if those adults have extensive developmental trauma and loss, as well as a history of out-of-home care.

In future longitudinal studies, consideration must be given to more holistic measures of function, with emphasis placed on the importance of assessing sleep. Sleep quality could play critical mediating and moderating roles for the subgroup of young adults who age out of care and struggle to adapt to adulthood [38,39]. It is also a condition that is less stigmatizing to discuss for some adults than a direct conversation about mental health. Therefore, this article also highlights the fact that identifying sleep challenges presents an important potential port of entry when connecting adults with care experiences to clinical services.

Life course well-being in foster care alumni is largely unexplored, and this gap in knowledge limits our understanding of developmental trajectories, reducing our ability to think through the developmental implications of our interventions. Therefore, this paper is a call to arms for health practitioners and researchers to track sleep more routinely during points of contact with foster youth and care leavers alike. We could track these adults’ well-being beyond their transition to adulthood.

### 4.1. Clinical Implications

An implication of this exploratory work is that sleep quality in populations who have had exposure to the child welfare system should be more routinely screened or asked about in clinical settings. Sleep is one area that is amenable to intervention, and the identification of sleep problems could also present a port of entry into gaining therapeutic insights about unresolved loss or trauma. Therefore, routine screening for sleeping difficulties in child welfare system-experienced people could be helpful [40]. Psychoeducation around sleep hygiene could be offered by many front-line professionals.

Sleep hygiene broadly encompasses practices and habits that contribute to a healthy sleep routine [41]. For example, a fundamental recommendation for sleep hygiene is to encourage those with sleeping challenges to go to bed and wake up at around the same time every day [42,43]. Creating a conducive sleep environment by trying to make a cool space with low light and turning off screens early can also be helpful [41]. Discussing the use of common stimulants during the day, such as caffeine and nicotine, and working with adults experiencing sleeping challenges to reduce the usage of these or other substances could also help improve sleep [41,44]. While it is beyond the scope of this article to highlight every aspect of sleep hygiene, these are skills that front-line intervention workers could pick up to improve their practice.

For people experiencing trauma-related nightmares, or hyperarousal, therapeutic integrations such as trauma-informed cognitive behavioural therapy [45,46], EMDR [47], or music relaxation may help to alleviate distressing sleeping experiences [48]. For adults who adopt healthy sleeping routines and still struggle significantly with their sleep, a medical consultation with a sleep specialist may be warranted. Many short- and long-term interventions are available to support those with sleeping difficulties, but which interventions are most effective for care leavers needs to be explored in future research.

### 4.2. Limitations

This study carries certain limitations that warrant consideration. The sample is a convenience sample comprising participants who were sourced from online care leaver networks. This non-representative sampling approach does not capture the full spectrum of experiences and identities within the broader population of North American care leavers. Our sample, active in the care leaver network community, also appears to have higher education than is typical for a care leaver population. While we suspect that our sample leans toward higher-functioning care leavers, as many were connected to care leaver networks, if that assumption holds true our findings likely represent a conservative estimate of the level of sleep problems in the broader care leaver population. The retrospective nature of the study and the challenges inherent in recruiting alumni of group/congregate care later in life limited our sample size and our ability to address causality and explore the direction of effects in more developmentally nuanced ways. While associations between youth protection experiences and sleep quality were identified, we are not in a position with this work to make causal inferences, and the temporal sequencing remains unclear. A cohort study using a larger sample than our smaller exploratory group would allow for regression model approaches that would be less vulnerable to the influence of outliers, potential model overfitting, and limited power. Longitudinal research designs would provide more robust findings, allowing us to explore potential amplifying reciprocal transactions between sleep and well-being, which are likely to be bidirectional in nature, over time. Future work would also benefit from sampling more specifically within cohort bands and stages of the life course. Given the challenges in recruiting those with group care experiences, we included age cohorts ranging from middle to later life, a wide range that is marked by shifts in sleep and behavioural patterns. More narrowly focused spans of the life course would allow for a fuller elucidation of the unique developmental needs and pathways within those periods of life. Lastly, the study’s qualitative component, while offering valuable insights into individual experiences, was not designed to investigate sleep directly. As a result, the narratives shared by the participants do not comprehensively capture the intricacies of the sleep challenges faced by all participants in this study. They merely point out that sleep challenges are complex for care leavers and should be investigated in a more representative sample in the future.

## 5. Conclusions

This study represents the first exploration, to our knowledge, into the sleep quality of child welfare system alumni aged 30+, shedding light on the often-overlooked intersection of adverse childhood experiences and adult sleep. Our findings underscore the enduring impact of negative care experiences on sleep across the lifespan. Our study suggests that incorporating routine inquiries about sleep into assessments in health and social service settings, particularly assessments of care leavers, could provide a valuable entry point for intervention. By recognizing, documenting, and addressing the enduring consequences of disrupted sleep among care leavers, we highlight the need for more targeted longitudinal work on this topic in this population, paving the way for more effective and targeted interventions, thereby enhancing the well-being and life trajectories of this vulnerable population.

## Figures and Tables

**Table 1:** Demographics.

Category	Subcategory	Count (%)
Age Distribution	31–40	16 (39%)
	41–50	9 (22%)
	51–60	6 (15%)
	61–70	5 (12%)
	71 or older	5 (12%)
Gender	Man	16 (39%)
	Woman	23 (56%)
	Non-binary or Third Gender	2 (5%)
Racial Identity	White	30 (73%)
	Racialized or Indigenous	10 (24%)
	Not Specified	1
Parental Status	Not a Parent	8 (20%)
	Parent	29 (70%)
	Not Disclosed	4 (10%)
Marital Status	Married	17 (41%)
	Widowed	2 (5%)
	Divorced	6 (15%)
	Separated	2 (5%)
	Never Married	14 (34%)
Education	Some High School	7 (17%)
	High School Diploma	3 (7%)
	GED	1 (2%)
	Some College or Trade School	4 (10%)
	Community College or Associate	5 (12%)
	Bachelor’s Degree	10 (25%)
	Master’s Degree	11 (27%)
	PhD or MD	0 (0%)
Intergenerational Placement	First Generation	26 (63%)
	Parent/Grandparent History	9 (22%)
	Third Generation	4 (10%)
	Unreported	2 (5%)
Time in Out-of-Home Care	1–2 Years	6 (15%)
	3–4 Years	8 (20%)
	5–7 Years	12 (29%)
	7–10 Years	5 (12%)
	10–15 Years	7 (17%)
	15+ Years	3 (7%)

**Table 2:** Predictors of current sleep quality in group care alumni.

		Models		
Variables	(1)	(2)	(3)	(4)
Quality of Placement	0.505 ** (0.219)		0.421 ** (0.221)	0.549 ** (0.284)
Friendship Support Network		0.360 * (0.088)	0.274 ^±^ (0083)	0.233 (0.104)
Age				−0.104 (0.428)
Gender				0.186 (0.849)
Race				0.116 (0.956)
Hispanic				−0.077 (2.33)
Education				−0.027 (0.222)
Annual Income				−0.001 (0.211)
Constant Adjusted R square	2.901 *** 0.24	3.152 ** 0.11	1.775 ** 0.26	1.188 0.23

^±^ *p* < 0.07, * *p* < 0.05, ** *p* < 0.01, *** *p* < 0.001.

**Table 3:** Predictors of current overall health problems in group care alumni.

		Models		
Variables	(1)	(2)	(3)	(4)
Sleep	−0.451 ** (0.345)		−0.279 ^±^ (0.325)	−0.328 * (0.286)
Friendship Support Network		−0.579 *** (0.181)	−0.477 ** (0.187)	−0.599 *** (0.188)
Age				−0.268 (0.691)
Gender				0.141 (1.518)
Race				0.058 (1.676)
Hispanic				0.168 (4.110)
Education				0.159 (0.375)
Annual Income				−0.065 (0.365)
Constant Adjusted R square	22.37 *** 0.18	24.18 *** 0.32	26.23 *** 0.37	20.67 ** 0.54

^±^ *p* < 0.06, * *p* < 0.05, ** *p* < 0.01, *** *p* < 0.001.

## Data Availability

Due to the privacy concerns the data supporting the findings of this research study are not publicly available. However reasonable requests for data sharing will be considered. Further inquiries can be directed to the corresponding author.
